# CXCL12-CXCR4 axis in feline mammary carcinoma

**DOI:** 10.18632/aging.101344

**Published:** 2017-12-11

**Authors:** Fernando Ferreira

**Affiliations:** Center for Interdisciplinary Research in Animal Health, Faculty of Veterinary Medicine, University of Lisbon, 1300-477 Lisbon, Portugal

**Keywords:** Feline mammary carcinoma, CXCL12, CXCR4, target therapy, HER2-positive subtype, cancer model

Chemokines and its specific receptors are known to be involved in the interplay between tumor microenvironment and cancer cells, modulating cellular proliferation, stemness maintenance, epithelial-mesenchymal transition and metastatic potential, as the immune response. In humans, the binding of the chemokine ligand C-X-C motif chemokine ligand 12 (CXCL12), also named as stromal cell-derived factor 1 (SDF-1), to the G-proteincoupled seven-transmembrane-domain chemokine receptor 4 (CXCR4), activates multiple signaling cascades (e.g. phosphatidylinositol-3-kinase/protein kinase AKT, extracellular signal-regulated kinase 1/2 and mitogen-activated protein kinase pathway), regulating tumor growth and metastasis of multiple cancer types, including liver, lung, bone, brain, prostate, ovarian, cervical, colorectal or pancreatic tumors [1]. In addition, the dysregulation of the CXCL12-CXCR4 signaling axis has also been linked to breast cancer development and progression, with organs and tissues showing CXCL12 overexpression found to be the most frequent metastatic sites (e.g. lung and liver). Furthermore, the expression of CXCL12 by stromal cells and tumorassociated macrophages, promotes tumor growth through autocrine and paracrine mechanisms [2]. In parallel, patients showing CXCR4 overexpression in primary breast lesions were associated with a higher incidence of metastases in lymph nodes and a decreased overall survival [2]. Regarding this scenario, targeted therapies against CXCL12-CXCR4 axis may be useful to treat cancer, with anti-CXCL12 aptamers, CXCR4 antagonists and anti-CXCR4 monoclonal antibodies been developed and tested in clinical trials to treat leukemia, lymphoma, colorectal, pancreatic and breast tumors. Recently, a clinical study showed that two CXCR4 inhibitors significantly reduced growth of HER2-positive breast tumors, including Herceptin and Docetaxel-resistant tumors, strongly suggesting that CXCR4 inhibition may be an efficient strategy to improve breast cancer treatment [3].

In cat, the mammary carcinoma is the third most common malignancy, sharing many clinical and pathological features with the human breast cancer, and therefore being considered a suitable model for comparative oncology studies [4]. However, so far, only limited data is available about the role the CXCL12- CXCR4 axis in feline mammary carcinoma (FMC), with two studies revealing that CXCR4 is overexpressed in primary tumors and has a proliferative effect in FMC immortalized cell lines [5,6]. Recently, we demonstrated that serum CXCL12 levels can be used as a biomarker to diagnose the feline mammary carcinoma (cut-off value ≥ 2 ng/ml), being able to discriminate HER2-overexpressing tumors from other tumor subtypes (cut-off value ≥ 4 ng/ml) [7].

**Figure 1:**
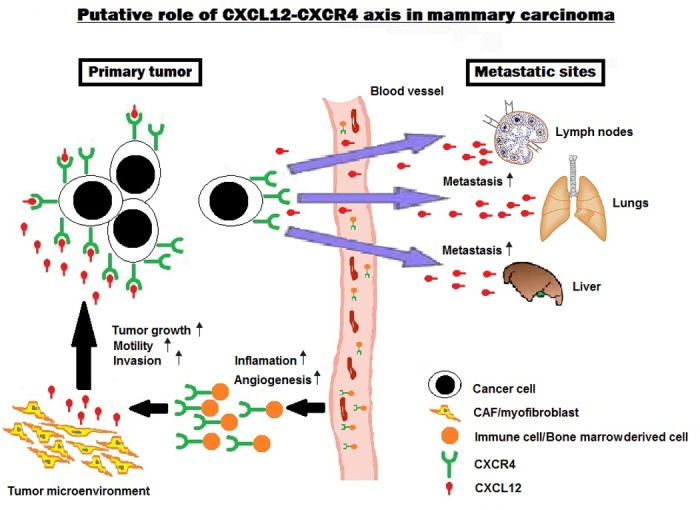
The putative roles of CXCL12-CXCR4 axis in feline mammary carcinoma Tumor cells expressing CXCR4 promote metastatic spread to organs showing overexpression of CXCL12 (e.g. lymph node, lungs, liver). In addition, the secretion of the ligand CXCL12 by tumor cells promotes the activation of CXCR4-CXCL12 axis locally, enhancing the primary tumor growth and inflammatory response. Finally, the production of CXCL12 by the tumor microenvironment (cancer-associated fibroblasts and myofibroblasts) enhances tumor cell mobility and invasion, and guides the recruitment of immune and bone marrow derived cells into the tumor microenvironment.

Considering the needing of new diagnostic tools and treatment approaches to improve the poor prognosis of cats with mammary carcinoma, and the relevant role of CXCL12-CXCR4 axis in human breast cancer, more studies on CXCL12 and CXCR4 expression in primary lesions, regional metastasis and distant metastasis (e.g. lungs, liver) are necessary in cat. Additionally, studies on associations between serum CXCL12 levels and CXCL12 status in metastasis will contribute to understand the role of this ligand in the metastatic process and if the role of the CXCL12-CXCR4 axis is conserved between human and cat (Figure 1). Finally, studies relating CXCL12 and CXCR4 expression to specific FMC subtypes (in particular with HER-positive and triple negative) and to prognostic factors (tumor size, overall and disease-free survival), will also corroborate that FMC is a suitable cancer model for comparative oncological studies.
